# Shortened Time to Identify *Staphylococcus* Species from Blood Cultures and Methicillin Resistance Testing Using CHROMAgar

**DOI:** 10.1155/2009/636502

**Published:** 2009-01-11

**Authors:** Shingo Chihara, Mary K. Hayden, Eileen Minogue-Corbett, Kamaljit Singh

**Affiliations:** ^1^Department of Medicine, Section of Infectious Diseases, Rush University Medical Center, 1653 W. Congress Parkway, Chicago, IL 60612, USA; ^2^Department of Pathology and Medicine, Section of Infectious Diseases, Rush University Medical Center, 1653 W. Congress Parkway, Chicago, IL 60612, USA; ^3^Department of Microbiology, Rush University Medical Center, 1653 W. Congress Parkway, Chicago, IL 60612, USA

## Abstract

The ability to rapidly differentiate coagulase-negative staphylococcus (CoNS) from *Staphylococcus aureus* and to determine methicillin resistance is important as it affects the decision to treat empiric antibiotic selection. The objective of this study was to evaluate CHROMagar *S. aureus* and CHROMagar MRSA (Becton Dickinson) for rapid identification of *Staphylococcus* spp. directly from blood cultures. Consecutive blood culture bottles (BacT Alert 3D SA and SN, bioMérieux) growing gram-positive cocci in clusters were evaluated. An aliquot was plated onto CHROMagar MRSA (C-MRSA) and CHROMagar *S. aureus* (C-SA) plates, which were read at 12 to 16 hours. C-SA correctly identified 147/147 *S. aureus* (100% sensitivity); 2 CoNS were misidentified as *S. aureus* (98% specificity). C-MRSA correctly identified 74/77 MRSA (96% sensitivity). None of the MSSA isolates grew on C-MRSA (100% specificity). In conclusion, CHROMagar is a rapid and sensitive method to distinguish MRSA, MSSA, and coagulase-negative *Staphylococcus* and may decrease time of reporting positive results.

## 1. Introduction

*Staphylococcus* species are the most commonly isolated bacteria from blood cultures [1–3]. The ability to rapidly differentiate coagulase-negative staphylococcus (CoNS) from *S. aureus* is important as CoNS is a common skin contaminant whereas *S. aureus* bacteremia is associated with significant morbidity and mortality [2, 3]. In addition, the increased prevalence of methicillin resistance in *Staphylococcus* spp. has led to the widespread use of vancomycin as empiric therapy, which may provide selective pressure for the spread of vancomycin-resistant enterococcus and the emergence of vancomycin-intermediate *S. aureus* (VISA) and vancomycin-resistant *S. aureus* (VRSA) [4]. The ability to rapidly differentiate CoNS from *S. aureus* and to determine methicillin susceptibility, particularly from blood cultures, is clearly desirable as it would allow more directed antibiotic therapy and limit the unnecessary use of vancomycin.

The objective of this study was to evaluate CHROMagar *S. aureus* (Becton Dickinson, Sparks, Md, USA) and CHROMagar MRSA (Becton Dickinson, Sparks, Md, USA) for rapid identification and susceptibility testing of *Staphylococcus* spp. directly from blood culture broth media.

## 2. Materials and Methods

The study was performed in the microbiology laboratory at Rush University Medical Center in Chicago (Ill, USA) from November 2006 to August 2007. Consecutive blood culture bottles (BacT/Alert 3D SE and SN bottles, bioMérieux, Durham, NC, USA) growing gram-positive cocci in clusters were evaluated. Only one set of blood cultures from each patient was included. A 0.05 mL aliquot of blood broth was plated onto CHROMagar MRSA (C-MRSA) and CHROMagar *S. aureus* (C-SA) plates that were divided into 4 quadrants to allow each plate to be used for 4 patient isolates. Plates were incubated at 35°C in ambient air. Growth on plates was evaluated after 12 to 16 hours of incubation and again after the manufacturer's recommended incubation time of 24 hours. Mauve colonies on C-SA plate were classified as *S. aureus*. Mauve colonies on C-MRSA were classified as MRSA. CoNS produced white colonies.

Results were compared to conventional microbiologic methods for identifying *Staphylococcus* spp., that is, gram stain, catalase reaction, and tube or slide coagulase testing (BactiStaph, Remel Inc. Lenexa, Kan, USA). Discrepant identification results were resolved by an automated identification method (Microscan Walkaway, Dade Behring Inc., West Sacramento, Calif, USA). Susceptibility testing was done by using the cefoxitin disk test according to CLSI guidelines [5] Methicillin resistance in *S. aureus* was confirmed by the detection of PBP2a using the MRSA screen slide agglutination test (Denka Seiken Co, LTD, Tokyo, Japan).

## 3. Results

Two hundred and sixty three unique blood culture broths growing gram-positive cocci in clusters were studied. Five bottles that yielded *Micrococcus* spp. were excluded from further analysis; none of these produced mauve colonies on C-MRSA or C-SA plates. There were 147 *S. aureus* and 111 CoNS identified by conventional microbiologic methods. C-SA correctly identified 147/147 *S. aureus* after 16 hours of incubation (100% sensitivity). There were 2 CoNS isolates that produced faint mauve colonies on C-SA; these were confirmed BactiStaph negative, and were identified as *Staphylococcus hominis* and *Staphylococcus haemolyticus*. Therefore, the specificity of C-SA for identification of *S. aureus* was 98%. The positive predictive value of the C-SA plates for identification of *S. aureus* was 99% (147/149) while the negative predictive value was 100% (109/109).

C-MRSA correctly identified 74/77 MRSA (96% sensitivity) after 12 to 16 hours incubation. None of the MSSA or CoNS isolates grew on C-MRSA (100% specificity). Both C-SA and C-MRSA plates were read again after 24 hours incubation; the results were completely concordant with those obtained after 12 to 16 hours incubation (data not shown). The positive predictive value of the C-MRSA plates for identification of MRSA was 100% (74/74) while the negative predictive value was 98% (181/184).

## 4. Discussion

Our study shows that the direct subculture of positive blood culture broths onto C-MRSA and C-SA plates allows for the rapid identification of *S. aureus* and determination of methicillin susceptibility. It is important to distinguish among MSSA, MRSA, and CoNS as management changes depending on the species isolated. It is a common practice among clinicians to initiate empiric vancomycin therapy when informed of a positive blood culture growing gram-positive cocci in clusters. The use of a beta-lactam antibiotic is preferred therapy if MSSA is isolated but current methods of susceptibility testing using oxacillin or cefoxitin disks require cultures to be held for 24 hours before susceptibility results can be reported. Hence, a decision to continue, stop, or change therapy is generally not possible in the first 24 to 48 hours of culture incubation using conventional microbiology methods. In addition, it is often recommended to place patients with MRSA infection on contact isolation as soon as possible; hence the rapid identification of MRSA is also an important infection control consideration.

CHROMagar *S. aureus* is a selective and differential medium developed to isolate and identify *S. aureus*. It has been used to identify *S. aureus* in clinical specimens such as respiratory samples and wound swabs [6, 7]. CHROMagar MRSA is a selective and differential medium developed to detect MRSA in nasopharyngeal specimens and thereby ascertain MRSA carriage [8]. However, there are few data on the use of these plates as primary media for subcultures of positive blood culture bottles. In a study by Pape et al., only C-MRSA was utilized and the plates were read at 18 to 24 hours. This study showed that the medium was highly sensitive (97.6%) and specific (99.9%) for identifying MRSA from blood culture broths [9]. In a similar study by Colakoglu et al., MRSA ID chromogenic medium (Becton Dickinson, Sparks, Md, USA) was used to identify MRSA directly from blood culture bottles, wound swabs, and abscesses. For blood cultures, the sensitivity was 97.8% and the specificity was 99.7% at 24 hours incubation [10]. We elected to use both CHROMAgar C-SA and C-MRSA as we feel that it is important to be able to rapidly identify both MSSA and MRSA bacteremia. We read C-SA and C-MRSA plates at 12 to 16 hours rather than at 18 to 24 hours of incubation as we surmised that there are significantly higher organism loads in blood culture broths compared to nasal swab specimens and most positive blood cultures are pure cultures compared to the mixed flora seen from nasal swabs. There was no discrepancy between the 12- to 16-hour and 24-hour readings.

While there are alternative methods to rapidly identify *Staphylococcus* spp., such as real-time PCR, they are more expensive, require specialized equipment and sometimes batch testing of specimens [11]. Direct tube coagulase test is a simple, inexpensive, and rapid method to test for coagulase-positive and coagulase-negative *Staphylococcal* spp. in blood culture broths, but the sensitivity of the test has been reported as low as 65% [12, 13]. In our study, the direct tube coagulase test had a high specificity (100%), but a sensitivity of only 71% (data not shown).

In the current study, there were 2 false-positive C-SA cultures that grew CoNS and these were identified as *S. hominis* and *S. haemolyticus*. Previous reports have suggested that other coagulase-negative *Staphylococcus spp.* including *S. cohnii, S. intermedius,* and *S. schleiferi,* may yield a faint mauve color on C-SA that with experience can be distinguished from the deep mauve color of *S. aureus* [14]. We suggest performing a gram-stain and coagulase test when there is a question as to the final identification of the organism isolated.

While none of the MSSA isolates produced mauve colonies on C-MRSA (negative predictive value 100%), there were three false negative MRSA isolates which did not give mauve colonies on C-MRSA (positive predictive value 98%). This suggests that while a positive result by C-MRSA can be reported as MRSA, a negative result should only be reported as *S. aureus* with susceptibility to be followed by conventional testing.

In conclusion, CHROMagar is a rapid and sensitive method to distinguish MRSA, MSSA, and CoNS directly from blood culture bottles. It is an easy method that can fit into the routine workflow of most clinical microbiology laboratories and that does not require any new or expensive equipment. Use of C-SA and C-MRSA may facilitate a decreased time for reporting positive results for MSSA and MRSA bacteremia.
